# Cervical rotation, chest deformity and pelvic obliquity in patients with spinal muscular atrophy

**DOI:** 10.1186/s12891-020-03710-7

**Published:** 2020-11-07

**Authors:** Agnieszka Stępień, Łucja Mazurkiewicz, Katarzyna Maślanko, Witold Rekowski, Maria Jędrzejowska

**Affiliations:** 1 Faculty of Rehabilitation, Józef Piłsudski University of Physical Education, Marymoncka 34, 00-968 Warsaw, Poland; 2 2nd Department of Neurology, Institute of Psychiatry and Neurology, Warsaw, Poland; 3 Center of Functional Rehabilitation ORTHOS, Warsaw, Poland; 4 Rare Diseases Research Platform, Mossakowski Medical Research Centre, Polish Academy of Sciences, Pawińskiego 5, 02-106 Warsaw, Poland

**Keywords:** SMA, Cervical rotation, Chest deformity, Pelvic obliquity, Scoliosis, Assessment

## Abstract

**Background:**

Musculoskeletal disorders are often observed in patients with spinal muscular atrophy (SMA). The aim of the study was to assess passive ranges of rotation in the cervical spine, chest deformity and pelvic obliquity in SMA patients, and to compare these results to the norms obtained in the group of healthy individuals. The second aim was to review these measurements and Cobb angle values for correlations in SMA patients.

**Methods:**

The study included 74 patients with SMA and 89 healthy individuals aged 2 to 18 years. Cervical Rotation (CR), Supine Angle of Trunk Rotation (SATR) and Pelvic Obliquity (PO) tests were carried out.

**Results:**

Cervical rotation ranges were significantly higher in the control group than in SMA patients (*p* < 0.05). Differences between cervical rotation ranges to the left and to the right were significantly larger in SMA I and SMA II groups than in healthy individuals (*p* = 0.000). Chest asymmetry and pelvic obliquity were bigger in SMA groups than in the control (*p* < 0.05). Significant correlations between cervical rotation measurements, chest deformity, pelvic obliquity and Cobb angle were found in SMA individuals, depending on the type.

**Conclusions:**

The results of the study suggest that CR, SATR and PO tests may assist in the assessment of SMA patients in addition to the radiographic evaluation of the spine. Biomechanical relationships between disorders located in various skeletal structures should be taken into account in the treatment of SMA patients. Special attention should be given to assessing postural parameters in non- sitters and sitters. Treatment of patients with SMA and associated musculoskeletal disorders requires a multi-specialist approach.

## Background

Spinal muscular atrophy (SMA) is a progressive neurodegenerative disease of an autosomal recessive trait of inheritance. In its course, motoneurons of the spinal cord are lost, which leads to the generalized muscle weakness and limited motor function [1–5]. The disease is characterized by great variability of the age of onset (from fetal age to adulthood) and severity of clinical symptoms (from lethal to asymptomatic). According the classification various clinical forms are distinguished: patients with prenatal onset (SMA 0, death within weeks), very week children unable to sit unaided (SMA I, the acute form), non-ambulant patients able to sit independently (SMA II, the intermediate form), ambulant patients, who may lose ability to walk (SMA III, mild form)) and ambulant adults (SMA IV,; onset of symptoms in adulthood) [6, 7].

Spinal muscular atrophy may lead to many complications: musculoskeletal (scoliosis, chest deformities, joint contractures, hip dislocation), respiratory (impaired cough reflex, respiratory distress), gastrointestinal (gastro-esophageal reflux, swallowing problems, malnutrition or obesity) and many others, depending on the type and stage [8–15].

Scoliosis, defined by the Scoliosis Research Society as a three dimensional deformation of the spine with the Cobb angle at least 10^0^ [16], occurs most often in SMA I, SMA II patients, as well as in individuals with type III who lost their ability to walk independently [7, 14, 16].

**Fig. 1:**
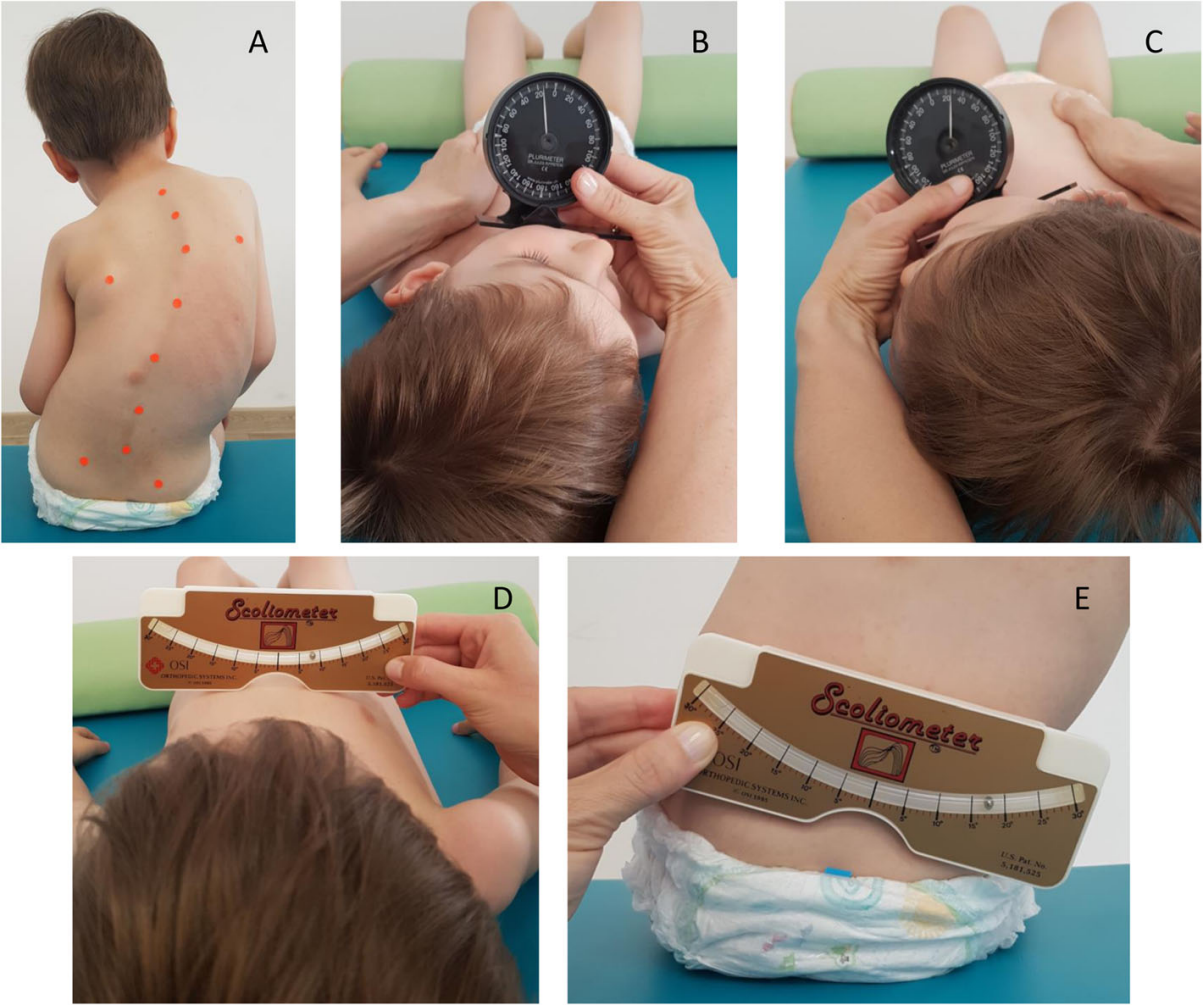
CR, SATR and PO tests in a boy with type SMA II and scoliosis. Note: **a** An improper head position, chest deformity and pelvic obliquity in a boy with type SMA II and scoliosis. **b** Cervical Rotation test to the right, **c** Cervical Rotation test to the left, **d** Supine Angle of Trunk Rotation Lower test, **e** Pelvic Obliquity test

The spinal deformity is usually accompanied by lateral flexion and rotation of the cervical spine and head, chest deformity and pelvic obliquity [8, 13, 17–20] (Fig. 1a). These musculoskeletal impairments may affect the daily functioning of patients with SMA. An incorrect cervical and head alignment may lead to limitations in cervical spine mobility or difficulties in swallowing [11, 21]. Distortion of the rib cage, associated with scoliosis, contributes to respiratory disorders occurring in SMA patients [9, 10, 20, 22], while pelvic obliquity may affect the ability to maintain balance in a sitting position [13, 17, 20].

Physiotherapy is an important part of the management of spinal muscular atrophy. Conducting effective physiotherapy is connected with the necessity to assess regular the musculoskeletal system and motor function [22]. It is particularly significant in view of screening programs introduced in some countries and new therapeutic procedures [6, 23, 24], since the application of new pharmacotherapies in SMA will probably change the whole natural history of the disease and significantly increase the number of patients with a chronic type of the disease.

A radiological examination, recommended at least once a year, is a standard in detecting or confirming skeletal deformities in patients with neuromuscular diseases [22, 25].

So far, no other additional tests have been commonly used in the assessment of the spinal deformities in SMA patients. The Adam’s test used in screening to diagnose idiopathic scoliosis [16, 26, 27], cannot be usually used in SMA individuals due to the limited ability to perform a forward bending in the standing or sitting position.

In our study we assessed the cervical spine mobility, chest shape and pelvis position with the use of reliable tests adapted to the functional state of SMA patients: Cervical Rotation test (CR), Supine Angle of Trunk Rotation test (SATR), and Pelvic Obliquity test (PO). The tests, performed with Rippstein’s plurimeter (Rippstein, Switzerland) and scoliometer (Orthopedic Systems Inc. OSI 1995), have been originally assessed in previous studies in the group of 30 patients aged 4–15 with type II and III SMA. The tests manifested excellent intraobserver and interobserver reliability [28].

The aim of the study was to assess passive ranges of rotation in the cervical spine, chest deformity and pelvic obliquity in SMA patients, and to compare these results to the norms obtained in the group of healthy individuals. The second aim was to review these measurements and Cobb angle values for correlations in SMA patients.

## Methods

The study was conducted after obtaining the acceptance of the Senate Research Ethics Committee at University of Physical Education in Warsaw (SKE 01–03/2016). The protocol was explained to all patients. The legal guardians of the participants gave their written consent to participate in the study.

### Subjects

The study included patients of both sexes aged 2 to 18 with SMA type I, II and III, diagnosed in a genetic examination and not treated pharmacologically. Individuals who underwent scoliosis surgery or tracheostomy, with spine pain or those with scoliosis without an actual X ray of the spine were not qualified for the study.

Healthy individuals of both sexes aged 2 to 18, without scoliosis and other diseases, were recruited as the control group. The Adam’s forward bending test [26] was used as initial assessment qualifying for a control group. Individuals with the value of the angle of trunk rotation at 5 degrees or more were not qualified for the study. Children and adolescents after injuries within the last year or reporting pain in the cervical, thoracic or lumbar spine were also not included in the control group.

### Protocol

The study was conducted during the conference and workshops for SMA patients and their families organized by SMA Foundation and during individual consultations in a physiotherapy centre. The examinations were performed by three physiotherapists familiar with problems of SMA patients. Prior to the study, the physiotherapists had been informed of the study protocol and trained how to perform measurements. The study protocol conducted in SMA and control groups included three tests: Cervical Rotation test, Supine Angle of Trunk Rotation test and Pelvic Obliquity test.

Cervical Rotation test (CR) – the measurement of the range of cervical spine rotation on the left (CR left) and right (CR right) side of the body in a supine position performed with the use of Rippstein’s plurimeter (Rippstein, Switzerland), zeroed parallel to the surface and placed on the corpus mandibulae [28] (Fig. 1b, c).

Supine Angle of Trunk Rotation test (SATR) – the measurement of chest deformity performed in a supine position with the use of a scoliometer (Orthopedic Systems Inc. OSI 1995). The scoliometer was set across the chest in the upper part of the sternum at the level of the 2nd-3rd rib (SATR Upper – SATRU) and in a lower part of the sternum (SATR lower – SATRL) (Fig. 1d) [28].

Pelvic Obliquity test (PO) – the measurement of pelvic obliquity was performed with the use of the scoliometer placed at the posterior superior iliac spines in a sitting position with supported lower limbs [28] (Fig. 1e). The measurement was performed in SMA II and SMA III individuals who were able to maintain a sitting position without assistance during the examination.

### Statistical analysis

The results were analysed statistically with the use of IBM SPSS Statistics v.20 software. The means and standard deviations for CR, SATR and PO were calculated. Moreover, for each of the participants, the sum of CR left and CR right (cervical rotation sum – CRS) and the difference between CR left and CR right (cervical rotation difference – CRD) were calculated. The Shapiro Wilk test was used to assess normal distribution. In order to estimate differences between the groups the Mann–Whitney U test and the One -Way Anova were applied. When performing multiple post hoc comparisons, the Scheffe test was used, taking into account the correction for the number of comparisons performed. The Spearman correlation was used to analyse the correlation between CR, SATR, PO and Cobb angle values. The Pearson’s chi square and Cramer’s coefficient tests were applied as a measure of association between nominal variables of measurements and the occurrence of scoliosis in SMA patients. The level of significance was set at *p* ≤ 0.05. The correlation strength was interpreted as follows: < 0.3 negligible correlation, 0.3–0.5 low positive (negative) correlation, 0.5–0.7 moderate positive (negative) correlation, 0.7–0.9 high positive (negative) correlation, > 0.9 – very high positive (negative) correlation [29].

## Results

This study recruited 74 patients with types I, II, III SMA aged 2 to 18 (mean age 7.1 ± 4.3 years, weight 22.4 ± 10.6 kg, height 120.4 ± 19.5 cm, all SMA patients), including 40 girls and 34 boys. The group consisted of 21 individuals (9 girls and 12 boys) with type I SMA, 36 (21 girls and 15 boys) with type II SMA and 17 (10 girls and 7 boys) with type III SMA.

The study group included 21 participants who were unable to sit independently (21 SMA I), 44 non-ambulant participants who were able to sit unsupported (36 SMA II, 8 SMA III) and 9 ambulant participants (SMA III).

Thirty eight participants with SMA (51.4%) used non-invasive ventilation, and 36 did not have respiratory support (48.6%). Twenty six patients with SMA (35.1%) declared that they did not use support standing, and 48 practiced support standing or walked (64.9%).

The revealed incidence of scoliosis was 77% (57 participants) in the SMA group. The incidence of scoliosis in SMA I group was 90%, in SMA II – 83% and in SMA III – 47%. In 50 participants single scoliosis was observed (mean 32.4^o^ ± 16.0), while in 7 individuals double scoliosis occurred (thoracic mean 47.9^o^ ± 20.1, thoracic/lumbar mean 59.1^o^ ± 25.4).

The results were compared to the control group of 89 individuals, including 47 girls and 42 boys aged 2–18. The values of age, weight, height in SMA patients and the control group and information about scoliosis in SMA individuals are presented in Table 1.

**Table 1:** Mean values of age, weight, height, CR, SATR, PO measurements and Cobb angle in participants

	SMA I ***N*** = 21	SMA II ***N*** = 36	SMA III ***N*** = 17	Control ***N*** = 89
**Age (years)** ± **SD**	6.5 ± 5.0	7.4. ± 4.1	7.2 ± 4.0	6.8 ± 3.7
**Weight (kg)** ± **SD**	20.7 ± 11.1	23.7 ± 10.6	21.6 ± 10.3	26.0 ± 13.4
**Height (cm)** ± **SD**	116.6 ± 22.9	122.3 ± 17.9	121.1 ± 18.9	125.1 ± 23.4
**CR left (o)** ± **SD**	20.4 ± 10.7	22.5 ± 9.1	24.5 ± 9.4	29.9 ± 4.8
Min / Max	0.0 / 40.0	0.0 / 36.0	6.0 / 38.0	18.0 / 40.0
**CR right (o)** ± **SD**	21.9 ± 10.8	24.8 ± 8.3	23.9 ± 9.3	30.4 ± 4.8
Min / Max	2.0 / 44.0	5.0 / 40.0	6.0 / 38.0	20.0 / 42.0
**CRS (o)** ± **SD**	42.3 ± 14.4	47.3 ± 14.3	48.4 ± 17.4	60.3 ± 9.3
Min / Max	10.0 / 70.0	5.0 / 76.0	12.0 / 70.0	40.0 / 82.0
**CRD (o)** ± **SD**	11.7 ± 10.7	6.9 ± 7.6	4.6 ± 4.6	2.0 ± 1.9
Min / Max	0.0 / 38.0	0.0 / 32.0	0.0 / 11.0	0.0 / 8.0
**SATRU (o)** ± **SD**	6.3 ± 3.8	4.3 ± 4.2	2.4 ± 1.7	0.5 ± 0.9
Min / Max	0.0 / 14.0	0.0 / 15.0	0.0 / 5.0	0.0 / 3.0
**SATRL (o)** ± **SD**	5.6 ± 3.7	5.1 ± 4.0	2.7 ± 1.8	0.3 ± 0.6
Min / Max	0.0 / 15.0	0.0 / 15.0	0.0 / 7.0	0.0 / 3.0
**PO (o)** ± **SD**	NA	8.7 ± 6.4	3.6 ± 3.0	0.8 ± 1.0
Min / Max	NA	0.0 / 24.0	0.0 / 10.0	0.0 / 4.0
**Single scoliosis** **Cobb angle (o)**	ThL 32.2 ± 19.1 N = 17	ThL 33.5 ± 15.0 *N* = 25	ThL 29.0 ± 15.0 N = 8	no scoliosis
**Double scoliosis** **Cobb angle (o)**	Th 59.0 ± 4.2 ThL 51.0 ± 29.1 *N* = 2	Th 43.4 ± 22.7 ThL 62.4 ± 26.5 *N* = 5	no scoliosis	no scoliosis

Note: *SMA I* Spinal muscular atrophy type I, *SMA II* Spinal muscular atrophy type II, *SMA III* Spinal muscular atrophy type III, *CR left* Cervical rotation to the left, *CR right* Cervical rotation to the right, *CRS* Sum of CR left and CR right, *CRD* Difference between CR left and CR right, *SATRU* Supine angle of trunk rotation upper; *SATRL* Supine angle of trunk rotation lower; *PO* Pelvic obliquity, *NA* Not analysed, *SD* Standard deviation, (^o^) Degrees, *N* Number of participants, *NA* Not analyzed, *Th* Thoracic curve, *ThL* Thoracic/lumbar curve

### Cervical rotation (CR)

The highest CR left, CR right and CRS values were noted in healthy participants, while the lowest values were observed in SMA I group (Table 1). The ranges of CR left and CR right were significantly bigger in the control group than in patients with SMA type I, II and III. The values of CR left and CR right did not differ between the SMA I, SMA II and SMA III groups (Table 2).

**Table 2:** Comparison of the CR, SATR and PO values obtained in SMA I, II, III and control groups

	CR left (^o^)	CR right (^o^)	CRS (^o^)	CRD (^o^)	SATRU (^o^)	SATRL (^o^)	PO (^o^)
**SMA I vs control**	***p*** = 0.000*	***p*** = 0.000*	***p*** = 0.000*	***p*** = 0.000*	***p*** = 0.000*	***p*** = 0.000*	NA
**SMA II vs control**	***p*** = 0.000*	***p*** = 0.001*	***p*** = 0.000*	***p*** = 0.000*	***p*** = 0.000*	***p*** = 0.000*	***p*** = 0.000*
**SMA III vs control**	***p*** = 0.044*	***p*** = 0.009*	***p*** = 0.003*	*p* = 0.363	*p* = 0.172	***p*** = 0.021*	***p*** = 0.011*
**SMA I vs SMA II**	*p* = 0.855	*p* = 0.798	*p* = 0.723	***p*** = 0.025*	*p* = 0.672	*p* = 0.994	NA
**SMA I vs SMA III**	*p* = 0.512	*p* = 0.931	*p* = 0.631	***p*** = 0.002*	***p*** = 0.003*	***p*** = 0.023*	NA
**SMA II vs SMA III**	*p* = 0.855	***p*** = 0.997	***p*** = 0.984	*p* = 0.559	***p*** = 0.026*	***p*** = 0.004*	***p*** = 0.000*

Note: *CR* Cervical rotation, *CR left* Cervical rotation to the left, *CR right* Cervical rotation to the right, *CRS* Sum of CR left and CR right, *CRD* Difference between CR left and CR right, *SATR* Supine angle of trunk rotation, *SATRU* Supine angle of trunk rotation upper, *SATRL* Supine angle of trunk rotation lower, *PO* Pelvic obliquity, *SMA I* Spinal muscular atrophy type I, *SMA II* Spinal muscular atrophy type II, *SMA III* Spinal muscular atrophy type III, *NA* Not analyzed, (^o^) Degrees, p- level of significance; * - significant difference

The value of CRS in the whole SMA group was approximately 20% lower than the value obtained in the control group. CRS was significantly smaller in all SMA subgroups compared to the groups of healthy individuals. The values obtained in SMA I, II and III groups did not differ significantly (Table 2).

The smallest differences between CR left and CR right were recorded in the control group. In 39 SMA participants, a difference of up to 5^o^ was noted between CR left and CR right (CRD), while in 35 individuals (47.2%) the difference was higher than 5^o^. Mean value of CRD in the whole SMA group differed significantly from the value in the control group (*p* < 0.05). The largest CRD values were noted in the SMA I and SMA II groups. The value of CRD in these two groups was significantly higher than in the control group. No significant differences between SMA III and control group were observed. However, a significant difference was noted between SMA I group and participants with type II and III (Table 2).

### Supine angle of trunk rotation (SATR)

In the majority of SMA patients, chest asymmetry was noted. Only in the case of 7 children with SMA II and SMA III (9.5% of the whole SMA group), the value of SATRU and SATRL measured with the scoliometer was 0^o^, while in the control group, a symmetrical chest was noted in 50 of the participants (56.2%). The most severe deformities of the chest were observed in patients with SMA I and SMA II, while the smallest in patients with SMA 3.

The values of SATRU in the control group were significantly lower than the values obtained in SMA I and SMA II groups. The values of SATRL obtained by the healthy participants were significantly lower than the values noted in SMA I, SMA II and SMA III groups.

Differences between SMA I, SMA II and SMA III groups were significant, with the exception of SATRU in SMA I and SMA II groups (Table 2).

### Pelvic obliquity (PO)

The PO test was performed on individuals with SMA II, SMA III and in the control group. All patients with SMA II and SMA III qualified for the tests were able to maintain the sitting position during the measurements. The lowest PO value was noted in controls, while the highest value was observed in patients with SMA II (Table 2). Pelvic obliquity occurred in 35 (97%) patients with SMA II and in 15 participants with SMA III (88.2%). The value of pelvic obliquity was higher than 4^o^ (the maximum PO value in the control group) in 24 (66.7%) participants with type II SMA. Only 3 persons with SMA II and SMA III (5.6%) had a symmetrical pelvis position (0^o^). In the case of 26 participants with SMA II and SMA III (49.1%), pelvic obliquity ranged between 0^o^ and 4^o^, while in 27 patients (50.9%) it was bigger than 4^o^. In the control group, a proper pelvis position was noted in 37 individuals (56.1%). In the remaining participants, obliquity was between 1^o^ and 4^o^.

Pelvic obliquity was significantly higher in SMA II and SMA III groups than in the control group. Also, a significant difference in pelvic obliquity between SMA II and SMA III groups was noted (Table 2).

### CR, CRD, CRS, SATR and PO parameters in SMA group depending on age

**Fig. 2:**
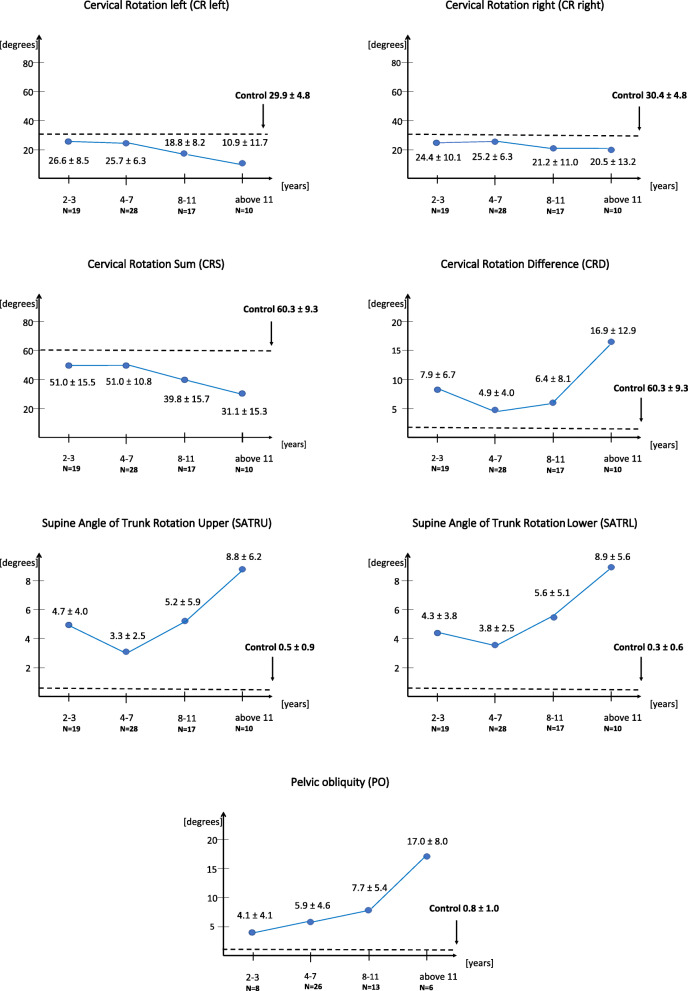
Variability of CR, SATR and PO tests values depending on age in the SMA group. Note: SMA – spinal muscular atrophy; (o) – degrees

The analysis of the values of cervical rotation, chest deformity and pelvic obliquity revealed that CR left, CR right and CRS values decreases, while chest deformity and pelvic obliquity increase in participants older than 8 years. The highest CRD and PO values were noted in participants above 11 years (Fig. 2).

### CR, SATR, PO values in SMA patients with scoliosis and without scoliosis

In 57 SMA participants with scoliosis, chest deformity (SATRU: Cramer’s V = 0.619, *p* = 0.000; SATRL: Cramer’s V = 0.678, *p* = 0.000) and oblique pelvic position (Cramer’s V = 0.811, *p* = 0.000) were significantly more frequent than in the subgroup without scoliosis (17 participants).

**Fig. 3:**
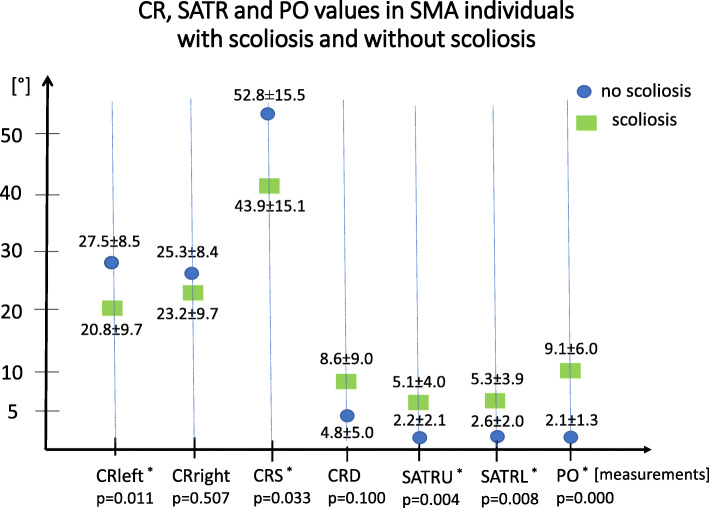
Comparison of CR, SATR and PO values in SMA individuals with scoliosis and without scoliosis. Note: CR – cervical rotation; CR left – cervical rotation to the left; CR right – cervical rotation to the right; CRS – sum of CR left and CR right; CRD – difference between CR left and CR right; SATR – supine angle of trunk rotation; SATRU – supine angle of trunk rotation upper; SATRL – supine angle of trunk rotation lower; PO – pelvic obliquity; SMA – spinal muscular atrophy; (o) – degrees; p- level of significance; * - significant difference

The analysis showed significant differences between CRleft, CRS, SATRU, SATRL and PO values obtained by SMA patients with scoliosis and without scoliosis. No differences between CRright and CRD were observed (Fig. 3).

### Correlations between CRD, CRS, SATR, PO and cobb angle in SMA groups

Due to differences in motor capabilities, the correlations between the parameters CR, SATR, PO and Cobb angle were tested separately in SMAI, SMA II and SMA III groups.

A significant low correlation between Cobb angle and CRD in the SMA I group was found. As the scoliosis angle increased, the difference between the cervical rotation ranges increased. In addition, a moderate relationship between SATRL and CRD was observed in this group. A low positive correlation relationship between chest asymmetry and CRD was found in the SMA II group, but it was not observed in the SMA III patients (Table 3).

**Table 3:** Correlations between CR, SATR, PO and Cobb angle values in SMAI, SMA II and SMA III groups

**SMA I (*N* = 21)**								
	**CRleft**	**CRright**	**CRS**	**CRD**	**SATRU**	**SATRL**	**PO**	**Cobb (*N*** **= 19)**
**CRleft** **p**	NA	−.059 .801	**.686**^a^ **.001**	−.216 .346	−.086 .710	−.162 .482	NA	−.440 .059
**CRright** **p**	−.059 .801	NA	**.628**^a^ **.002**	−.083 .720	−.106 .647	−.207 .368	NA	.031 .900
**CRS** **p**	**.686**^a^ **.001**	**.628**^a^ **.002**	NA	−.188 .414	−.161 .486	−.281 .218	NA	−.290 .229
**CRD** **p**	−.216 .346	−.083 .720	−.188 .414	NA	.282 .215	**.514**^a^ **.017**	NA	**.460**^a^ **.048**
**SATRU** **p**	−.086 .710	−.106 .647	−.161 .486	.282 .215	NA	.389 .081	NA	.248 .307
**SATRL** **p**	−.162 .482	−.207 .368	−.281 .218	**.514**^a^ **.017**	.389 .081	NA	NA	.360 .130
**PO**	NA	NA	NA	NA	NA	NA	NA	NA
**Cobb** **p**	−.440 .059	.031 .900	−.290 .229	**.460**^a^ **.048**	.248 .307	.360 .130	NA	NA
**SMA II (*N* = 36)**								
	**CRleft**	**CRright**	**CRS**	**CRD**	**SATRU**	**SATRL**	**PO**	**Cobb (*N*** **= 30)**
**CRleft** **p**	NA	.304 .071	**.830**^a^ **.000**	.033 .848	−.010 .953	.116 .502	−.177 .301	−.310 .095
**CRright** **p**	.304 .071	NA	**.729**^a^ **.000**	.284 .093	.234 .170	.128 .456	.217 .203	−.196 .299
**CRS** **p**	**.830**^a^ **.000**	**.729**^a^ **.000**	NA	.142 .408	.031 .857	.065 .705	−.044 .799	−.335 .070
**CRD** **p**	.033 .848	.284 .093	.142 .408	NA	**.445**^a^ **.006**	**.494**^a^ **.002**	**.416**^a^ **.012**	.257 .171
**SATRU** **p**	−.010 .953	.234 .170	.031 .857	**.445**^a^ **.006**	NA	**.578**^a^ **.000**	**.391**^a^ **.018**	.321 .084
**SATRL** **P**	.116 .502	.128 .456	.065 .705	**.494**^a^ **.002**	**.578**^a^ **.000**	NA	**.464**^a^ **.004**	**.404**^a^ **.027**
**PO** **p**	−.177 .301	.217 .203	−.044 .799	**.416**^a^ **.012**	**.391**^a^ **.018**	**.464**^a^ **.004**	NA	**.703**^a^ **.000**
**Cobb** **p**	−.310 .095	−.196 .299	−.335 .070	.257 .171	.321 .084	**.404**^a^ **.027**	**.703**^a^ **.000**	NA
**SMA III (*N* = 17)**								
	**CRleft**	**CRright**	**CRS**	**CRD**	**SATRU**	**SATRL**	**PO**	**Cobb (N = 8)**
**CRleft** **p**	NA	**.713**^a^ **.001**	**.928**^a^ **.000**	.402 .110	.038 .884	−.155 .552	**−.655**^a^ **.004**	−.313 .451
**CRright** **p**	**.713**^a^ **.001**	NA	**.907**^a^ **.000**	.408 .104	−.037 .887	−.131 .618	−.243 .346	.042 .921
**CRS** **p**	**.928**^a^ **.000**	**.907**^a^ **.000**	NA	.371 .143	−.059 .821	−.197 .449	**−.509**^a^ **.037**	−.024 .955
**CRD** **p**	.402 .110	.408 .104	.371 .143	NA	.265 .304	.142 .588	−.077 .770	.307 .459
**SATRU** **p**	.038 .884	−.037 .887	−.059 .821	.265 .304	NA	.189 .467	.075 .776	.123 .772
**SATRL** **p**	−.155 .552	−.131 .618	−.197 .449	.142 .588	.189 .467	NA	.051 .847	−.111 .793
**PO** **p**	**−.655**^a^ **.004**	−.243 .346	**−.509**^a^ **.037**	−.077 .770	.075 .776	.051 .847	NA	.457 .255
**Cobb** **p**	−.313 .451	.042 .921	−.024 .955	.307 .459	.123 .772	−.111 .793	.457 .255	NA

Note: *CR* Cervical rotation, *CR left* Cervical rotation to the left, *CR right* Cervical rotation to the right, *CRS* Sum of CR left and CR right, *CRD* Difference between CR left and CR right, *SATR* Supine angle of trunk rotation, *SATRU* Supine angle of trunk rotation upper, *SATRL* Supine angle of trunk rotation lower, *PO* Pelvic obliquity, *SMA I* Spinal muscular atrophy type I, *SMA II* Spinal muscular atrophy type II, *SMA III* Spinal muscular atrophy type III, *N* Number of participants, *NA* Not analyzed, (^o^) Degrees; ^a^ - significant correlation

The analysis showed a high correlation between Cobb angle and PO in SMA II group. Larger Cobb angle values ​​corresponded to larger pelvic tilt values. There was no such association in SMA III group. PO measurements in SMA II group were also positively low correlated with CRD, SATRU and SATRL values. An moderate positive correlations between PO, CRleft and CRS parameters were observed in SMA III participants (Table 3).

### Scoliosis, non-invasive ventilation and support standing in SMA group

There was no significant relationship between the incidence of scoliosis and the use of non-invasive ventilation (V-Cramer coefficient = 0.082, *p* = 0.482). The comparison of the mean values of the Cobb angle in the participants using non-invasive ventilation (mean Cobb 27,4^0^) and not using ventilatory support (mean Cobb 45,2^0^) showed no significant differences (*p* = 0.076). The analysis of postural parameters indicated no significant differences between the values of CRleft (*p* = 0.080), CRD (*p* = 0.636), SATRU (*p* = 0.615), SATRL (*p* = 0.563) and PO (*p* = 0.369). In the ventilated patients, significantly higher values of CRright (*p* = 0.050) and CRS (*p* = 0.020) were found.

Low but statistically significant strength of the relationship between the incidence of scoliosis and support standing/ walking (V-Cramer coefficient = 0.335, *p* = 0.004) was noticed. Scoliosis occurred in 96.2% of people who did not declare spending their time standing. In the group of people who reported supported standing or walking, scoliosis occurred in 43.2%.

The participants who reported standing or walking presented less severe deformities of the upper chest SATRU (*p* = 0.043). The higher values of cervical rotation CRleft (*p* = 0.002), CRright (*p* = 0.004) and CRS (*p* = 0.001) were also observed in this group. There was no difference between the of SATRL (*p* = 0.068), CRD (*p* = 0.175) and PO (*p* = 0.084) parameters.

## Discussion

Spine and chest deformities may affect the functional state of individuals with neuromuscular diseases [13, 20, 21, 30]. Therefore, the international recommendations for diagnosis and management of SMA recognize the importance of a regular physical examination, with a focus on the musculoskeletal system. It is recommended that the physical examination should include, inter alia, assessment of the spine alignment, range of motions, muscle strength, motor functions and the quality of daily activities [22]. The assessment of young children enables early detection of deformities and implementation of appropriate interventions.

In our study it was revealed that there exist significant differences in cervical rotation, chest shape and pelvis position while sitting between SMA patients and healthy individuals.

It was observed that CR in SMA patients is limited compared to healthy individuals. Differences between the ranges of rotation to the left and to the right in SMA I and SMA II groups were significantly higher than in the control group. In the SMA I group, a significant positive correlation between CRD and Cobb’s angle was found. This means that in children and adolescents with higher values of Cobb angle, the difference between the ranges of left and right cervical rotation increased. We have not found any studies on cervical rotation in SMA patients. Previous studies examined only contractures of the upper and lower extremities [14, 15] and their impact on limitations in daily activities [31]. Salazar et al. found that minimal hip and knee joint contractures in patients with SMA types II and III were associated with diminished motor ability [31].

Difficulties in everyday functioning resulting from an improper head position, often related to scoliosis, are also rarely mentioned [20]. Some authors drew attention to a potential correlation between head position, body position and swallowing [11, 21]. The biggest limitation of the range of rotation in the cervical spine weas observed by us among the oldest participants. This restriction may be related to swallowing difficulties that occur in people with SMA, but this phenomenon requires further study. The important goals for rehabilitation in SMA patients is to prevent contractures and maintain function and mobility [22]. Therefore, it is worth introducing neck stretching exercises into therapy.

In our study the SATR test revealed that chest deformities in SMA patients occurred more often and were significantly severe than in the healthy individuals. The highest values of SATR were noted in individuals with SMA I and SMA II who most often experienced breathing difficulties and need non-invasive ventilatory support [32–34]. In the case of patients with SMA III, the test revealed significantly lower values of chest deformities compared to SMA I and SMA II participants. The abnormal, bell-shaped chest in patients with SMA types I and II was found in studies conducted with the use of the 3D opto-electronic plethysmography. SMA III patients participating in this study showed an almost rectangular ribcage shape [35].

One of the factors that can affect breathing in people with neuromuscular diseases is scoliosis [36]. A significant correlation between the severity of scoliosis and the percentage of predicted vital capacity and peak flow was found in SMA patients in previous studies [9]. The weakness of respiratory muscles and chest deformity, usually related to scoliosis, have been recognized as the main causes of breathing disorders [8–10, 12, 13]. The study by Fujak et al. [12] revealed that among 4–6-year-old patients with SMA type II, there was a distinct reduction in the relative vital capacity, while in SMA III patients relative vital capacity values were higher. Differences in the shape of the chest in the SMA subgroups demonstrated in our study may affect respiratory parameters, but this hypothesis needs to be confirmed in the future.

We found that pelvic obliquity in patients with SMA was significantly severe than in the control group. Moreover, the values of pelvic obliquity was higher in SMA II than in SMA III group. It has been highlighted in the literature that scoliosis and improper pelvis position constitute the main problems in orthopaedic treatment of the majority of SMA patients [13, 17]. On the basis of radiological images, Fujak et al. concluded that pelvic obliquity in a sitting position occurred in the majority of SMA II patients, regardless of age, i.e. even in children aged 0–4 years. They also noted that both scoliosis and pelvic obliquity increased in older participants and is more severe in SMA II than in SMA III patients [13]. Our observations confirm these results. Pelvic obliquity was observed in the majority of SMA II patients. In addition, the strongest correlations between PO and Cobb angle were observed in the this group. An asymmetrical pelvic position was found in 92% children with SMA II and SMA III aged 2–4 years. The obliquity of the pelvis increased in older participants.

The results obtained by us confirmed that age has a significant impact on the progression of skeletal deformities and limitations of range of motion. The values ​​of cervical rotation, chest deformity and pelvic obliquity were worse in older children. Deterioration of these parameters was observed in children over 7 years of age. Gradual progression of impairments in the musculoskeletal system may hypothetically affect negative changes of motor functions assessed by functional scales, that are observed especially in no-ambulant patients during the growth [37].

This study has another practical application due to the fact that it sought correlations between disorders located in various parts of the body in SMA patients. A significant correlations between the cervical rotation parameters, chest asymmetry and pelvic obliquity were found in the SMA subgroups. It suggests the need for constant monitoring of the cervical spine range of rotation, chest structure and the pelvic position in these patients.

The analysis carried out in SMA participants with scoliosis and without scoliosis showed significant differences between these groups. CR, SATR and PO measurements showed more severe disorders in SMA patients with scoliosis. In addition, a significant correlation was observed between the Cobb angle and CRD values in participants with the type SMAI, Cobb angle and thoracic deformity measurements, and pelvic obliquity in the SMA2 group. It indicates the relationship between the severity of scoliosis and the analyzed postural parameters in children, who were unable to walk. Therefore non-sitters and sitters should be carefully monitored, as also suggested by other authors [7, 37].

Higher values of the ranges of cervical rotation were found in individuals who reported using non-invasive ventilation and support standing/walking. Participants using support standing systems or walkers showed smaller deformation in the upper part of the chest. These preliminary observations confirm the accuracy of the recommendations regarding rehabilitation [22] and require further observation in larger groups of participants.

The presented study has some limitations. This project did not take into account the functional status of the participants. In the future, it is worth to analyze the correlation between the size of scoliosis, postural parameters and motor function assessed with validated scales.

This study has shown that CR, SATR and PO tests provide relevant information about body posture in SMA patients. Tests are easy to apply, safe and enable early detection of changes in the musculoskeletal system. The results indicate that there is a need to implement interventions aimed at increasing the range of cervical rotation, correction of the chest or positioning in patients with SMA. Measurements could be applied in the treatment process as an addition to the X-ray image, especially in patients currently being treated pharmacologically and to evaluate different therapeutic interventions.

A multidisciplinary approach is fundamental principle in the management of SMA patients [22]. The spine surgeons responsible for the treatment of neuromuscular scoliosis often have a significant impact on the overall treatment. The collected data and results entitle us to offer some suggestions for the spine surgeons and other specialists involved in the treatment of a child with SMA with coexisting musculoskeletal disorders. Due to the observed correlations between the severity of scoliosis, ranges of cervical rotation, chest deformity and pelvic oblique position, it is important to evaluate various structures of the musculoskeletal system and their functions while examining a person with SMA. Patients with SMA and coexisting musculoskeletal disorders or scoliosis should be referred to physiotherapists and other specialists who can implement stretching exercises, proper positioning, bracing or respiratory stimulation. Support standing and no-invasive ventilation have a positive effect on the shaping of the chest and cervical rotation. Non-ambulant patients should be monitored carefully and regularly.

## Conclusions

The results of the study suggest that Cervical Rotation (CR), Supine Angle of Trunk Rotation (SATR) and Pelvic Obliquity (PO) tests may assist in the assessment of SMA patients in addition to the radiographic evaluation of the spine. Biomechanical relationships between disorders located in various skeletal structures should be taken into account in the treatment of SMA patients. Special attention should be given to assessing postural parameters in non- sitters and sitters. Treatment of patients with SMA and associated musculoskeletal disorders requires a multi-specialist approach.

## Data Availability

Available from the corresponding author if requested (please contact orthosas@wp.pl).

## Abbreviations

SMA: Spinal muscular atrophy
CR: Cervical Rotation test
CR left: Cervical rotation to the left
CR right: Cervical rotation to the right
CRS: Sum of CR left and CR right
CRD: Difference between CR left and CR right
SATR: Supine Angle of Trunk Rotation test
SATRU: Supine Angle of Trunk Rotation Upper test
SATRL: Supine Angle of Trunk Rotation Lower test
PO: Pelvic Obliquity test
